# Spatiotemporal integration of visual stimuli and its relevance to the use of a divisional power supply scheme for retinal prosthesis

**DOI:** 10.1371/journal.pone.0228861

**Published:** 2020-02-21

**Authors:** Yueh-Chun Tsai, José Jiun-Shian Wu, Po-Kang Lin, Bo-Jyun Lin, Pin-Shiou Wang, Ching-Hsiang Liu, Chung-Yu Wu, Chuan-Chin Chiao

**Affiliations:** 1 Institute of Systems Neuroscience, National Tsing Hua University, Hsinchu, Taiwan; 2 Department of Life Science, National Tsing Hua University, Hsinchu, Taiwan; 3 School of Medicine, National Yang Ming University, Taipei, Taiwan; 4 Department of Ophthalmology, Taipei Veterans General Hospital, Taipei, Taiwan; 5 National Experimental High School at Hsinchu Science Park, Hsinchu, Taiwan; 6 Department of Electronics Engineering, National Chiao Tung University, Hsinchu, Taiwan; 7 Biomedical Electronics Translational Research Center, National Chiao Tung University, Hsinchu, Taiwan; Ecole Polytechnique Federale de Lausanne, SWITZERLAND

## Abstract

A wireless photovoltaic retinal prosthesis is currently being studied with the aim of providing prosthetic vision to patients with retinitis pigmentosa (RP) and age-related macular degeneration (AMD). The major challenge of a photovoltaic device is its limited power efficiency. Our retinal prosthetic design implements a unique divisional power supply scheme (DPSS) system that provides the electrical power generated by all of the solar cells to only a subset of electrodes at any moment in time. The aim of the present study was to systematically characterize the spatiotemporal integration performance of the system under various DPSS conditions using human subjects and a psychophysical approach. A 16x16 pixels LED array controlled by Arduino was used to simulate the output signal of the DPSS design, and human performance under different visual stimulations at various update frequencies was then used to assess the spatiotemporal capability of retinal prostheses. The results showed that the contrast polarity of the image, image brightness, and division number influenced the lower limit of the update frequency of the DPSS system, while, on the other hand, visual angle, ambient light level, and stimulation order did not affect performance significantly. Pattern recognition by visual persistence with spatiotemporal integration of multiple frames of sparse dots is a feasible approach in retinal prosthesis design. These findings provide an insight into how to optimize a photovoltaic retinal prosthesis using a DPSS design with an appropriate update frequency for reliable pattern recognition. This will help the development of a wireless device able to restore vision to RP and AMD patients in the future.

## Introduction

Numerous studies have demonstrated that visual information is preserved, for a short period of time at least, after a stimulus offset. This perceptual phenomenon of spatiotemporal integration is generally known as visual persistence [1]. For example, it has been shown that if two visual stimuli in which either of them by itself is a collection of random dots are displayed separately within a short time interval, the subjects can perceive the pattern of 3-letter nonsense syllable [2, 3]. Subsequently, other researchers have also examined the mechanisms of information persistence in various contexts [4–8]. For instance, it has been reported that object recognition is able to be achieved by temporally integrating partial shape cues that are a subset of dots that mark the outer boundary of nameable objects and this happens over several hundred milliseconds [5]. In a visual word recognition task, it has also been shown that when the odd and even letters of a string are displayed alternatively at around 80 ms of presentation duration, the word is easily fused into a single percept and normal reading is achieved [9].

**Fig 1:**
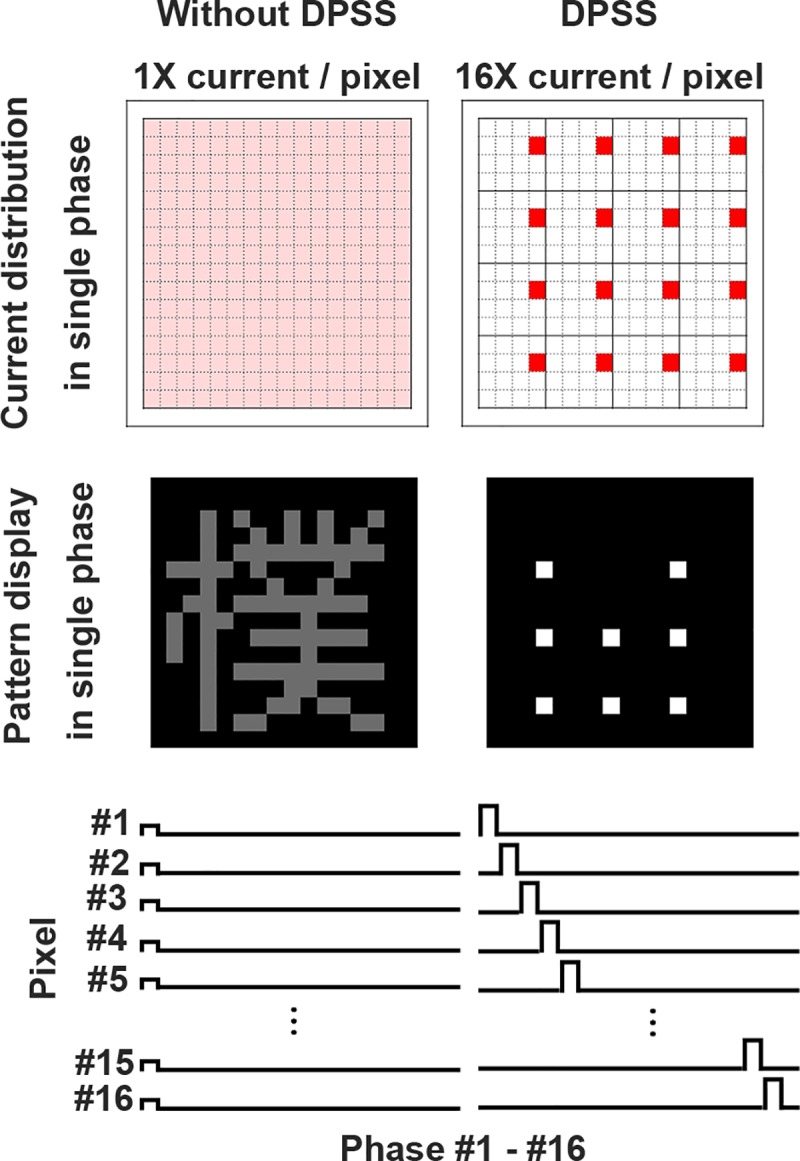
The concept of the divisional power supply scheme (DPSS). The configurations used for the standard system and the 16 divisions of the DPSS in terms of chip design. The electrical current distribution and the pattern display in a single phase are also shown for comparison.

The concept of visual persistence may have great potential as part of retinal prosthesis design, and this is the focus of the present study. Although there are several potential ways of restoring vision to individuals who suffer from retinitis pigmentosa (RP) and age-related macular degeneration (AMD), currently retinal prostheses are the only clinically tested treatment for RP and AMD patients. In a nutshell, a retinal prosthesis is designed to evoke neural activity by electrically stimulating retinal cells using a multiple electrode array. Despite the success of retinal prostheses recently, one of the major challenges in this field is the limit imposed by power efficiency during electrical stimulation [10]. To improve the visual acuity of a retinal prosthesis, there will inevitably be a demand for more electrodes within the array and this demand will continue to increase [11]. However, more electrodes mean that more power is needed. In addition, more power generates more heat, which will have a detrimental effect on the retinal prosthesis and surrounding tissue [12]. One way to resolve this issue, namely the power efficiency limitation, is to deliver the combined power obtained from many photovoltaic units to only a subset of electrodes at any moment in time, a so-called divisional power supply scheme (DPSS, see Fig 1) [13, 14]. Under such a system the charge release from electrical stimulation will be determined by the duration of the current pulse [15] and thus this approach, while generating adequate charge at each photovoltaic unit, will require a lower switch frequency of stimulating electrodes. However, in order to achieve normal visual perception, the switch frequency of stimulating electrodes within a sub-region must be sufficiently fast for visual persistence to take place. Thus, while the DPSS should be able to significantly increase the power efficiency of each electrode, the update frequency, the inverse of the duration for completing all the stimulating electrodes in each frame, needs to be characterized systematically for creating a successful retinal prosthesis.

Despite extensive research on visual persistence in the past, including the recognition of dot arrangements, geometric shapes, animal outlines, object outlines, and letters from the English alphabet [4, 6–8, 16], most studies have used only two alternating phases. In the design used for the DPSS described above, however, there are many more phases across the sparse display, for example sixteen phases in Fig 1; such a set needs to be integrated spatiotemporally in order to allow pattern recognition to take place. Similar ideas regarding a DPSS design for a retinal prosthesis have been investigated recently [17, 18]; however, the spatiotemporal integration performances of the various possible DPSS designs have not been systematically examined. In the present study, a visual psychophysics approach has been adapted in order to investigate visual persistence using a DPSS paradigm. The various parameters that affect the DPSS design, including pattern contrast, visual angle, ambient light level, image brightness level, flash order, and division number, were tested using human subjects to examine the lower limit of the DPSS update frequency. Although the major goal of this study was to search for the optimal DPSS parameters to be used in a retinal prosthesis for reliable visual function, the results of this study also serve as a basis for studying pattern recognition by spatiotemporal integration using multiple frames of sparse dots. Thus, this study also extends our understanding of visual persistence at multiple levels.

## Materials and methods

### Subjects

There were total of 17 subjects participated in the present study. Each of experiments was composed of 10 subjects. All subjects were native Chinese speaking students attending National Tsing Hua University and were aged between 20~35 years. All subjects performed a visual acuity test before the experiments started in order to ensure that they had 20/20 vision. The general nature of the tasks required and the protocols to be used were explained to subjects, but the specific stimulus conditions were not provided to them initially. All subjects were informed clearly and given their written consents before the experiment. This research adhered to the tenets of the Declaration of Helsinki. The experimental protocol was approved by the Research Ethics Committee of the National Tsing Hua University (REC number: 10603HM012).

### Experimental setup

The experiments were performed in a light-controlled room (85 or < 1 lux). The subjects placed with their head on a chin rest in order to keep the distance between the display board and the subject’s eyes constant. Four 8x8 LED arrays (1.8x1.8 cm each) were assembled to form a 16x16 LED array that acted as the display board. The diameter of each LED and the center-to-center distance between LEDs in the array were 1.9 mm and 2.5 mm, respectively. Each LED emits red light with a peak wavelength of 640 nm and an intensity of illumination around 8 mcd. The visual stimuli were controlled by two Arduino microcontroller boards (Arduino MEGA 2560 rev3) with a clock speed of 16 MHz. The display board was controlled by either a smart phone or PC with a custom written program that allowed the subject to start the experiment at will and to perform the task by choosing the correct image from all possible stimuli shown on the screen, except in one experiment that the subject must report the character verbally. The subject’s responses were recorded using the same program and then analyzed.

### Visual stimulus implemented by the divisional power supply scheme

The divisional power supply scheme (DPSS) circuit is designed to enhance the power supply of photovoltaic retinal prosthetics [13]. The concept involves the provision of electrical current alternately to a subset of electrodes at each phase using the electricity generated by all of the shared photovoltaic units. For example, in the sixteen divisions of a 16x16 multi-electrode (16x16 pixel) array, each electrode in a sub-region of a 4x4 array would obtain all the power from the sixteen shared pixels in this sub-region at each phase (Fig 1). The duration for completing each cycle of 16 phases depends on the update frequency. To implement the DPSS in the LED array as a visual stimulus, the on-or-off of one particular LED in a sub-region at each phase was determined based on the displayed visual stimulus pattern (Fig 1). The spatial order of the 16 LEDs’ on-or-off temporal sequence in a sub-region was specifically designed such that each pixel’s on-or-off was temporally separated from neighboring pixels in order to avoid interference or cross-talk in the photovoltaic retinal prosthetics.

### Experimental protocol

Subjects were required to stare at the center of the LED array at the start of each experiment (details described below), but they were allowed to freely move their eye gaze during the task. Before each experiment, the subject performed 40 practical trials with all possible visual patterns displayed at the highest update frequency, which are the easiest conditions. The aim of this step was to familiarize the subjects with the display board, the user program, and the visual stimuli. All experiments were composed of 200 trials. Each trial displayed one visual pattern for 1 second selected from a pool of four possible visual stimuli and ten possible DPSS update frequencies (1, 2, 3, 4, 5, 6, 8, 10, 16, and 20 Hz, the upper limit of our Arduino microcontroller board), and these were presented in a random order, except in one experiment in which the visual pattern was chosen from a pool of 210 non-repeated Chinese characters (S1 Fig). The subjects were required to choose one pattern out of four possible ones shown on the screen. Each visual pattern at each update frequency was shown five times, and thus there were total 200 trials (4 patterns x 5 repeats x10 update frequencies) that formed each of the four-alternative forced choice experiments, except in the orientation experiment, which was a two-alternative forced choice task and included also 200 trials (10 repeats). The response time was unlimited but recorded by the user program in some experiments, and the subjects proceeded with each trial at their own pace. After finishing each experiment, the subjects were required to rest for at least 5 minutes before they conducted the next experiment. Subjects conducted less than three experiments per day to avoid any performance decrease due to fatigue. They were usually able to complete the assigned tasks within 60 minutes.

### Experimental design

#### Experiment 1: Effect of pattern contrast

**Fig 2:**
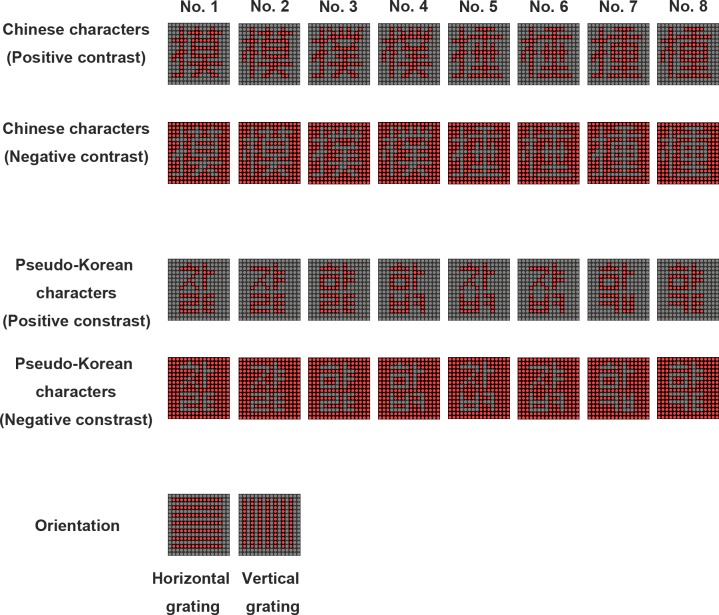
The visual stimuli used during the pattern recognition and orientation detection tasks. The pixelized images of the Chinese and Korean characters in positive and negative contrasts, and the gratings during the vertical and horizontal orientations. The mean numbers of total bright dots in each image are 68 and 190 in positive and negative contrasts, respectively (see Table 1 for details).

Eight real Chinese characters and eight pseudo Korean characters were used for this set of experiments (Fig 2). These Chinese and Korean characters were chosen such that the first four formed a group and the last four formed another group. Within each group, any one of the characters was different from the others by at least one half (either left or right for the Chinese characters and left, right, up, or down for the Korean characters), but they also resembled each other in appearance. Since all subjects were Chinese first language users and literate, the Chinese characters were treated as the words in the test. By way of contrast, none of the subjects knew Korean, thus they treated the Korean characters as simply patterns during the test. Due to the difference in the complexity between the Chinese and Korean characters, the numbers of pixels in the LED array representing these characters were different (Table 1). To minimize the learning effect in recognizing the Chinese and Korean characters, either, one of the first four characters (No. 1–4), or one of the last four characters (No. 5–8) was randomly chosen in the experiments. To further eliminate the decision bias resulting from seeing characters previously displayed at higher update frequency, in a separate experiment, the visual pattern was chosen from a pool of 210 non-repeated negative contrast Chinese characters (S1 Fig). To prevent the subjects from using certain pixels/areas in the LED array as a cue to recognize the characters, except for the pattern selected from a pool of 210 non-repeated Chinese characters, the visual pattern was displayed on any one of nine (3x3) possible locations by moving the pattern one or two pixels randomly. Both positive and negative contrasts of these characters were tested during the present study. It should be noted that the numbers of pixels in the LED array displaying positive and negative contrasts were also different (Table 1). The subjects performed these experiments in a dark room and the display board was placed at the distance that resulted in an 8° visual angle. In addition, the DPSS was set at 16 divisions (Fig 1) and the display of each phase was spatially separated.

**Table 1:** Parameters of the patterns displayed on the LED array.

Pattern	Width (dots)	Horizontal visual angle (degree) @ 25 cm / @ 100 cm	Height (dots)	Vertical visual angle (degree) @ 25 cm / @ 100 cm	Total dots (Positive / Negative contrast)
Chinese character #1	13	8.01 / 2.01	13	8.01 / 2.01	80 / 176
Chinese character #2	13	8.01 / 2.01	13	8.01 / 2.01	78 / 178
Chinese character #3	13	8.01 / 2.01	13	8.01 / 2.01	80 / 176
Chinese character #4	13	8.01 / 2.01	13	8.01 / 2.01	78 /178
Chinese character #5	13	8.01 / 2.01	13	8.01 / 2.01	76 / 180
Chinese character #6	13	8.01 / 2.01	13	8.01 / 2.01	74 / 182
Chinese character #7	13	8.01 / 2.01	13	8.01 / 2.01	84 / 172
Chinese character #8	13	8.01 / 2.01	13	8.01 / 2.01	82 / 174
Pseudo Korean character #1	9	5.15 / 1.29	13	8.01 / 2.01	50 / 206
Pseudo Korean character #2	9	5.15 / 1.29	13	8.01 / 2.01	51 / 205
Pseudo Korean character #3	9	5.15 / 1.29	13	8.01 / 2.01	59 / 197
Pseudo Korean character #4	9	5.15 / 1.29	13	8.01 / 2.01	54 / 202
Pseudo Korean character #5	9	5.15 / 1.29	13	8.01 / 2.01	47 / 209
Pseudo Korean character #6	9	5.15 / 1.29	13	8.01 / 2.01	48 / 208
Pseudo Korean character #7	9	5.15 / 1.29	13	8.01 / 2.01	54 / 202
Pseudo Korean character #8	9	5.15 / 1.29	13	8.01 / 2.01	55 / 201
Horizontal grating	13	8.01 / 2.01	13	8.01 / 2.01	91
Vertical grating	13	8.01 / 2.01	13	8.01 / 2.01	91
Chinese characters used in a non-repeated test (average)	15	9.23 / -	16	9.86 / -	- / 176 ± 0.68

#### Experiment 2: Effect of the viewing angle

**Fig 3:**
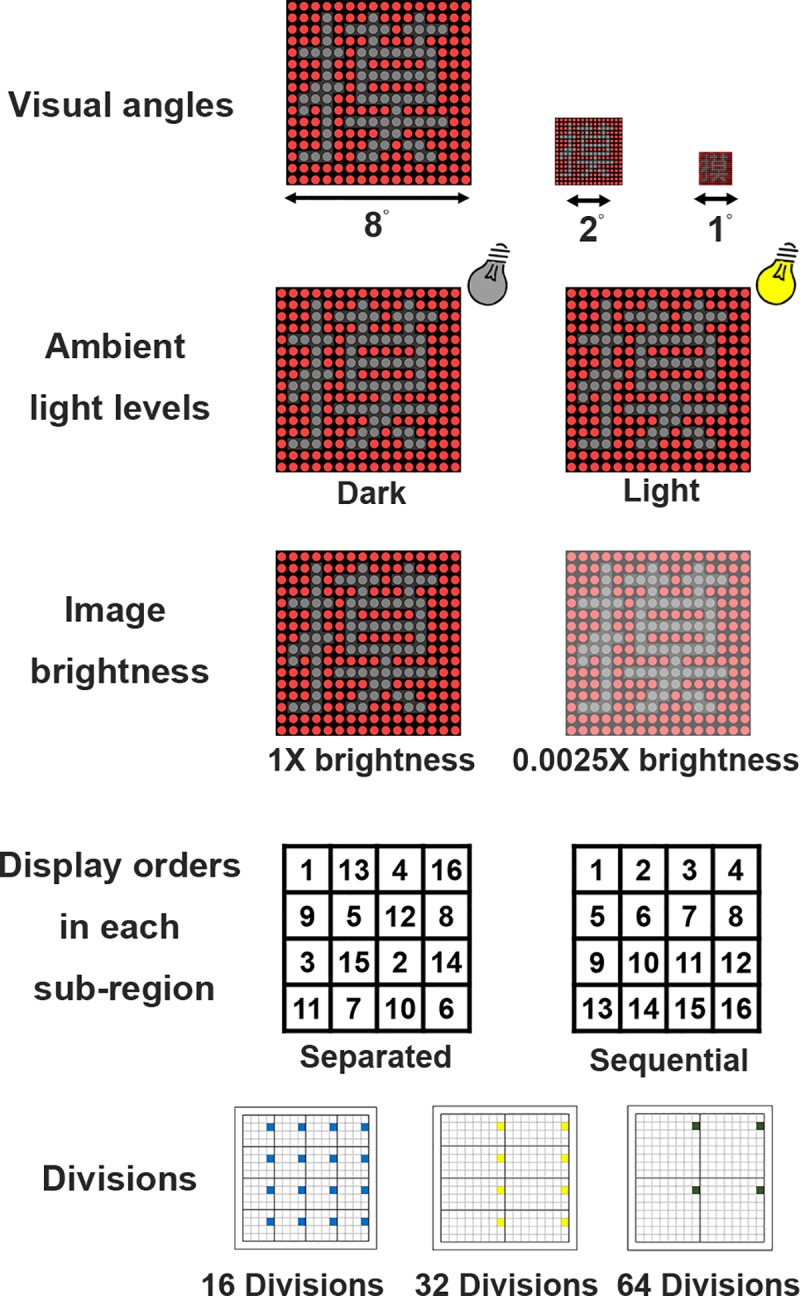
Conditions for visual stimuli used in all experiments. The different visual stimuli were carried out at various visual angles, ambient light levels, image brightness levels, display orders for each sub-region, and division numbers.

To examine the effect of the viewing angle on the recognition of the visual patterns during the DPSS mode, in addition to the Chinese and Korean characters (negative contrast) used in the Experiment 1, vertical and horizontal square wave gratings (2 pixel/cycle) were also included (Fig 2). The width and the number of pixels used in displaying these visual stimuli on the LED array are shown in Table 1. The display board was placed at the distance such that there was an 8° visual angle that covered both the central and peripheral vision areas or 2°/1° visual angles that covered only the central vision area (Fig 3). Subjects performed these experiments in a dark room and the DPSS was set at 16 divisions (Fig 1) using spatially separated phases.

#### Experiment 3: Effect of the ambient light level

To examine the effect of the ambient light level on the recognition of visual patterns in the DPSS mode, Chinese characters with negative contrast that were used in Experiment 1 were tested. The display board was placed in either a dark room or a well-lit room with a fluorescent lamp (60 W) on the ceiling (Fig 3). The level of illumination was 85 lux when the room light was on, and the level of illumination was below 1 lux when the light was off. Subjects were allowed to adapt to the ambient light level for 5 minutes before the experiment started. The light intensity of the 16x16 LED display board was identical at both ambient light levels. Subjects performed these experiments at an 8° visual angle, and the DPSS was set at 16 divisions (Fig 1) using spatially separated phases.

#### Experiment 4: Effect of the image brightness

To examine the effect of the image brightness on the recognition of visual patterns in the DPSS mode, Chinese characters with negative contrast that were used in Experiment 1 were tested. The ND400 neutral density filter (Green. L, China) was covered in front of the 16x16 LED display board to reduce the brightness to 0.25% of its original intensity (Fig 3). Subjects performed these experiments in a dark room at an 8° visual angle, and the DPSS was set at 16 divisions (Fig 1).

#### Experiment 5: Effect of flash order in a sub-region

To examine the effect of flash order in a sub-region on the recognition of visual patterns in the DPSS mode, Chinese characters with negative contrast were used as in the Experiment 1. The flash sequence of each pixel in a sub-region was either spatially separated or spatially sequential (Fig 3). Subjects performed these experiments in a dark room at an 8° visual angle, and the DPSS was set at 16 divisions (Fig 1).

#### Experiment 6: Effect of DPSS division number

To examine the effect of number of shared electrodes in a sub-region of the DPSS on the recognition of visual patterns, Chinese characters with negative contrast as used in the Experiment 1 were tested. In addition to the standard 16 divisions used in most experiments, a DPSS with 32 and 64 divisions (8 and 4 sub-regions in the 16x16 array, respectively) was also included (Fig 3). Subjects performed these experiments in a dark room at 8° visual angle, and the display of each phase in the DPSS was spatially separated.

### Threshold estimation and statistics

The hit rates obtained in each task were fitted to the psychometric function from previous study for estimating the threshold [19–21]. The Weibull psychometric function is:

$$y=\gamma +(1-\gamma -\lambda )∙\left\{1-\exp\left[-{\left(x/\alpha \right)}^{\beta }\right]\right\}$$ (1)

Where x is the update frequency, y is the hit rate obtained in each task, and γ is the guess rate (0.25 in the case of 4AFC; 0.5 in the case of 2AFC). α, β, and λ are free parameters: α and β affect the threshold and slope of the curve, respectively; λ corresponds to the miss rate. The data were fitted with the psychometric function using MATLAB program (The MathWorks Inc). The threshold was estimated by calculating the update frequency at 0.625 of the hit rate during both the 4AFC Chinese and Korean character recognition tests, at 0.75 of the hit rate during the 2AFC orientation discrimination test, and at 0.5 of the hit rate during the non-repeated pattern recognition test. The Wilcoxon signed rank test was used to determine whether there is a statistically significant difference between two conditions.

## Results

### Experiment 1: Effect of pattern contrast

**Fig 4:**
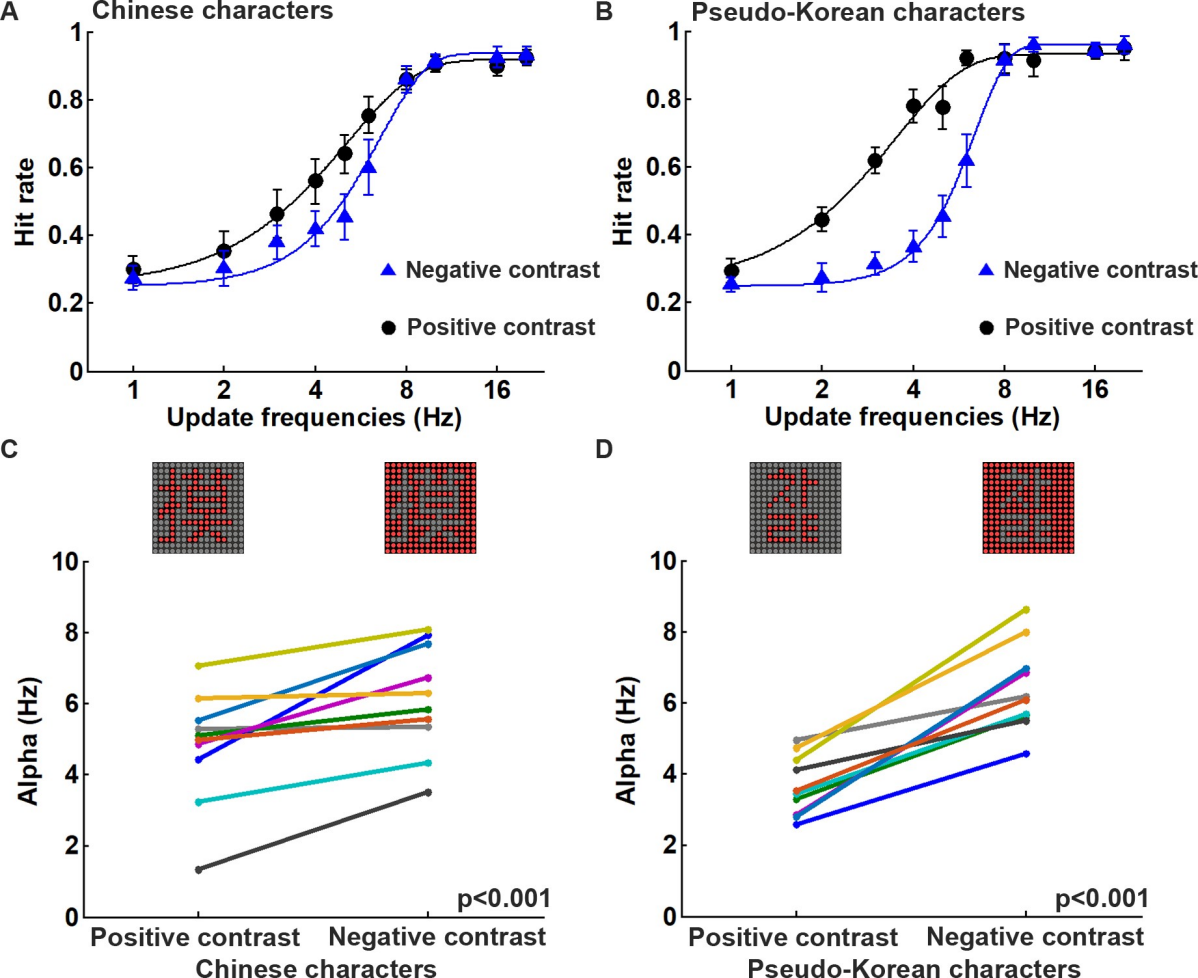
The performance during the positive and negative contrasts using DPSS is significantly different. (A)&(B) The mean hit rates and the standard errors at each update frequency are recorded for ten subjects during the image recognition tests of Chinese and Korean characters in either positive or negative contrast. The fitting curves are based on the Weibull function and are shown as black and blue lines, representing image recognition under positive and negative contrast conditions, respectively. Both experiments were performed in 8° visual angle, in a dark room, with a DPSS condition of 16 divisions and with spatially separated phases. (C)&(D) The comparisons of the alpha values obtained by fitting to the psychometric function in each subject for positive and negative contrasts in Chinese and Korean character recognition tests, respectively (Chinese characters, *p* < 0.001, n = 10; Korean characters, *p* < 0.001, n = 10).

To examine the effect of the pattern contrast on visual persistence, ten subjects performed the pattern recognition tasks using Chinese and Korean characters under both positive and negative contrast (Fig 2). It was found that recognizing the characters on the DPSS display in negative contrast was relatively more difficult than that in positive contrast at certain update frequencies for both the Chinese and Korean characters (Fig 4). It also appears that the psychometrical function in Chinese character recognition was different from that in Korean character recognition when the display was in negative contrast (blue curves in Fig 4). On average, subjects were able to recognize the Chinese characters with 62.5% hit rate at the update frequencies of 4.61 Hz and 6.02 Hz using positive and negative contrast, respectively (Fig 4A). However, the recognition threshold was 3.10 Hz and 5.97 Hz for the Korean characters in positive and negative contrast, respectively (Fig 4B). When the alpha values (point of subjective equality, or PSE), which were obtained by fitting the data using the psychometric function, were compared between the display patterns with positive and negative contrast (Fig 4C & 4D), it is clear that under negative contrast the patterns had significantly larger alpha values than under positive contrast, and this was true for both the Chinese and Korean character recognition tasks (Chinese character, *p* < 0.001; Korean character, *p* < 0.001; n = 10). These results support the hypothesis that visual persistence during pattern recognition is more challenging when the character is under negative contrast than when it is under positive contrast. Based on this finding, only the negative contrast patterns were used in the following experiments in order to examine the effects of the various DPSS parameters on visual persistence.

Although we have attempted to minimize the learning effect in recognizing the Chinese and Korean characters by randomly choosing one of the four characters selected from two sets (Fig 2) and presenting ten possible DPSS update frequencies in a random order, inevitably the subject might have seen a character at higher frequency before seeing the same character at lower frequency in the experiment. To further eliminate the decision bias resulting from seeing characters previously displayed at higher update frequency, in a separate experiment, the visual pattern was chosen from a pool of 210 non-repeated negative contrast Chinese characters (S1 Fig). On average, subjects were able to recognize the Chinese characters with 50% and 62.5% hit rates at the update frequencies of 7.62 Hz and 6.70 Hz using non-repeated and repeated patterns, respectively (S2A Fig). Note that the hit rates for estimating the threshold were different, 50% in the non-repeated pattern test and 62.5% in the repeated pattern, as the guess rates were different in two cases. When the alpha values were compared between the display with non-repeated and repeated patterns, there was no significant difference between the performance using repeated and non-repeated patterns (S2B Fig; *p* = 0.461; n = 10). This finding suggests that using repeated patterns in the present study would not result in a decision bias.

Despite the response time for subjects to make a decision in these psychophysics tests was unlimited, all subjects made choices in 2.42±0.9 sec. In selected experiments in which we have recorded the response time for each trial, it was found that subjects tended to make a fast decision at lower and higher update frequencies, whereas the response time increased at intermediate update frequencies near the threshold (S3 Fig). This finding suggests that the response time is a useful indicator of the difficulty of the task, and could be used a proxy for estimating the threshold or identify outliers.

### Experiment 2: Effect of viewing angle

**Fig 5:**
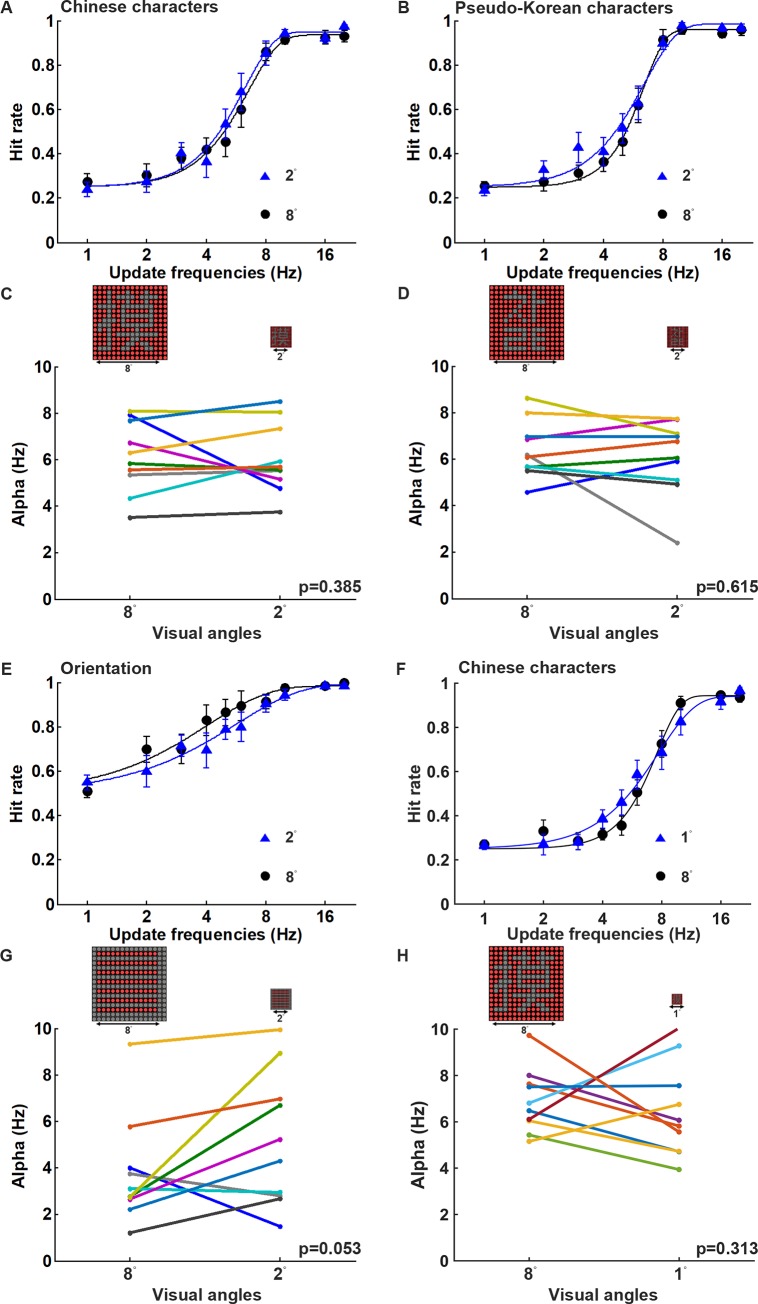
The performance at different visual angles using DPSS is not significantly different. (A)-(B) Average hitting rates and fitting curves at the different update frequencies for Chinese and Korean character recognition tasks at 2° and 8° visual angles, respectively. (C)-(D) The comparisons of the alpha values obtained by fitting of the psychometric function in each subject at 2° and 8° visual angles for Chinese and Korean characters, respectively (Chinese characters, *p* = 0.385, n = 10; Korean characters, *p* = 0.615, n = 10). (E) Average hitting rates and fitting curves at the different update frequencies for the orientation detection task at 2° and 8° visual angles. (F) Average hitting rates and fitting curves at the different update frequencies for Chinese characters recognition task at 1° and 8° visual angles. (G) The comparison of the alpha values obtained by fitting of the psychometric function in each subject at 2° and 8° visual angles for the orientation detection task (*p* = 0.053, n = 10). (H) The comparison of the alpha values obtained by fitting of the psychometric function in each subject at 1° and 8° visual angles for Chinese characters task (*p* = 0.313, n = 10). All experiments were performed in a dark room, and the DPSS condition was 16 divisions and with spatially separated phases.

To examine the effect of the pattern size on visual persistence, the display board was placed at the distance either an 8° visual angle, which covers both the central and peripheral vision, or 2°/1° visual angles, which cover only the central vision (Fig 3). The Chinese and Korean characters (negative contrast) used in the Experiment 1 and horizontal/vertical gratings (2 pixel/cycle) were included in this experiment. It was found that the viewing angle had little effect on character recognition and orientation detection tasks (Fig 5). On average, subjects were able to recognize the Chinese characters with 62.5% hit rate at the update frequencies of 5.68 Hz and 6.02 Hz when the visual angles were at 2° and 8°, respectively (Fig 5A). Similarly, subjects could recognize the Korean characters with 62.5% hit rate at the update frequencies of 5.66 Hz and 5.97 Hz when the visual angles were at 2° and 8°, respectively (Fig 5B). During the orientation detection task, subjects were able to discriminate vertical and horizontal gratings with 75% hit rate at the update frequencies of 4.37 Hz and 3.22 Hz when the visual angles were 2° and 8°, respectively (Fig 5E). To compare the recognition difference if the visual angle was further reduced to 1°, it was found that subjects were able to recognize the Chinese characters with 62.5% hit rate at the update frequencies of 6.97 Hz and 7.11 Hz when the visual angles were at 1° and 8°, respectively (Fig 5F). When the alpha values were compared between the display patterns at the two visual angles, it appears that there was no significant difference between 2° and 8° of visual angle for both the Chinese and the Korean character recognition tasks (Fig 5C & 5D; Chinese character, *p* = 0.385; Korean character, *p* = 0.615; n = 10), as well as during the orientation detection task (Fig 5G; *p* = 0.053; n = 10). Furthermore, there was also no significant difference between 1° and 8° of visual angle for the Chinese character recognition task (Fig 5H; *p* = 0.313; n = 10). These findings suggest that visual persistence during character recognition and orientation detection is independent of the pattern size when saccadic eye movement is allowed in the task.

### Experiment 3: Effect of ambient light level

**Fig 6:**
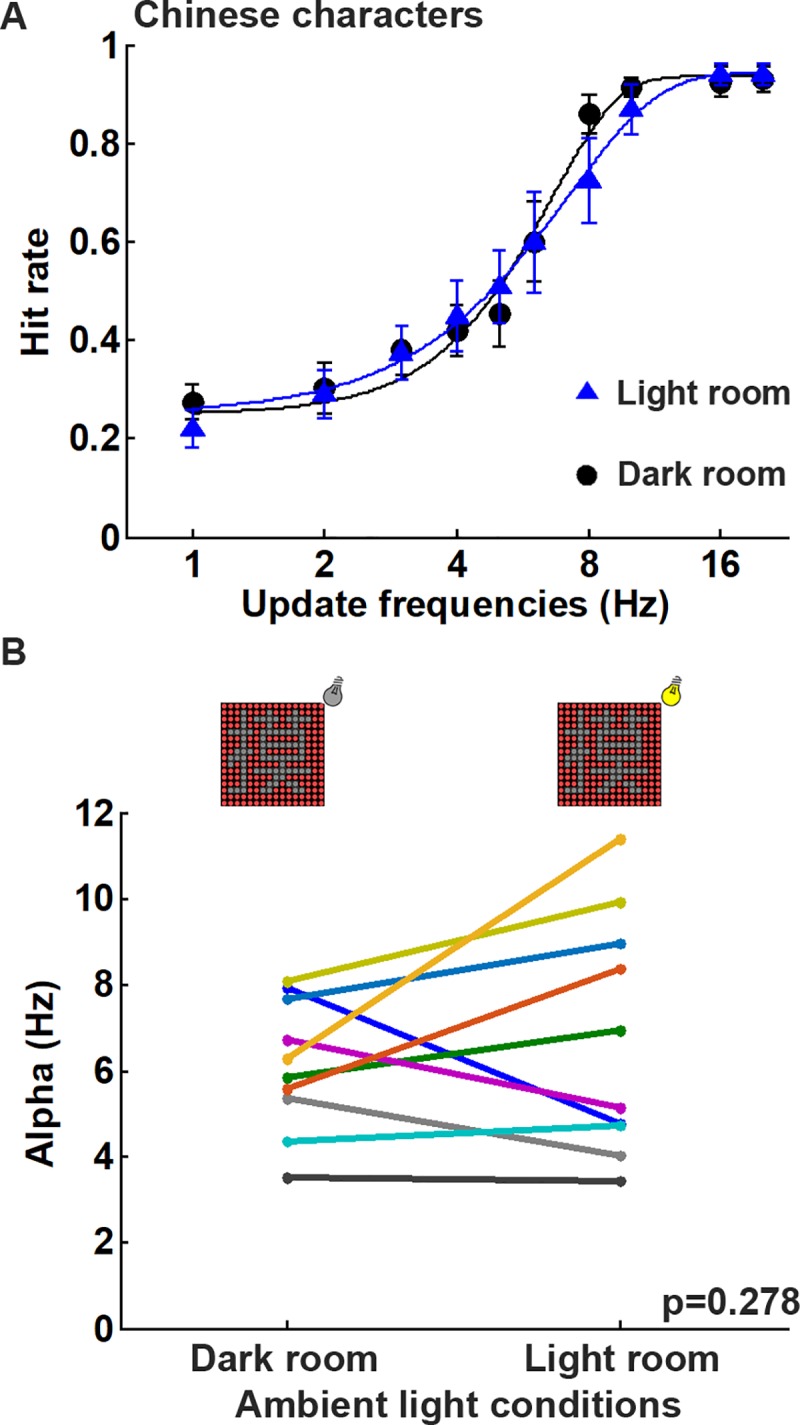
The performance at different ambient light levels using DPSS is not significantly different. (A) Average hitting rates and fitting curves at the different update frequencies in either a light or a dark room. The experiments were performed at 8° visual angle, and the DPSS condition was 16 divisions with spatially separated phases. (B) A comparisons of the alpha values obtained by fitting to the psychometric function for each subject showed no significant change in performance at different ambient light levels when carrying out the Chinese character recognition tasks (*p* = 0.278, n = 10).

To examine the effect of ambient light level on visual persistence, the Chinese character recognition task was conducted in a lit room (85 lux) and in a dark room (Fig 3). It was found that the ambient light level had little effect on the character recognition tasks (Fig 6). On average, subjects were able to recognize the Chinese characters with 62.5% hit rate at the update frequencies of 6.31 Hz and 6.02 Hz when the task was performed in the lit and dark rooms, respectively (Fig 6A). When the alpha values were compared between the display patterns at different ambient light levels (Fig 6B), there was no significant difference between the lit and dark rooms for the Chinese character recognition task (*p* = 0.278; n = 10). This result indicates that visual persistence during character recognition is not dependent on the ambient light intensity.

### Experiment 4: Effect of image brightness

**Fig 7:**
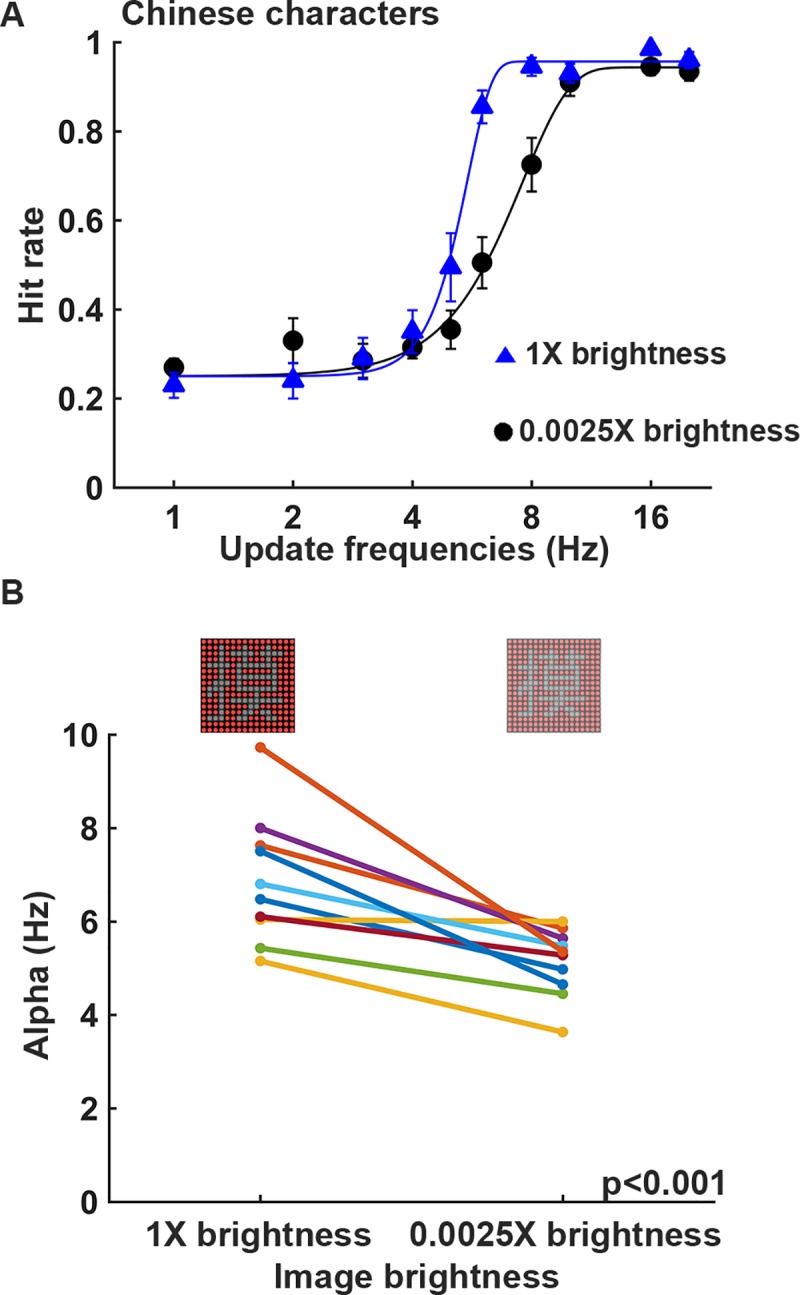
The performance at different display brightness levels using DPSS is significantly different. (A) Average hitting rates and fitting curves at the different update frequencies at the image brightness levels of 1X and 0.0025X. The experiments were performed in a dark room at 8° visual angle, and the DPSS condition was 16 divisions with spatially separated phases. (B) The comparison of the alpha values obtained by fitting to the psychometric function for each subject showed a significant difference in performance at different image brightness levels for the Chinese character recognition task (*p* < 0.001, n = 10).

To examine the effect of image brightness on visual persistence, the Chinese character recognition task was conducted with and without the ND400 neutral density filter covered in front of the 16x16 LED display board (Fig 3). It was found that recognizing the Chinese characters on the DPSS display with the ND400 filter was relatively easier than that without the ND400 filter at certain update frequencies (Fig 7). On average, subjects were able to recognize the Chinese characters with 62.5% hit rate at the update frequencies of 5.32 Hz and 7.11 Hz when the task was performed with and without the ND400 filter, respectively (Fig 7A). When the alpha values were compared between the display patterns at different image brightness (Fig 7B), the alpha values for the lower image brightness were significantly smaller than the alpha values for the original image brightness (*p* < 0.001; n = 10). This result indicates that the performance of Chinese character recognition is better on the lower brightness DPSS display.

### Experiment 5: Effect of flash order in a sub-region

**Fig 8:**
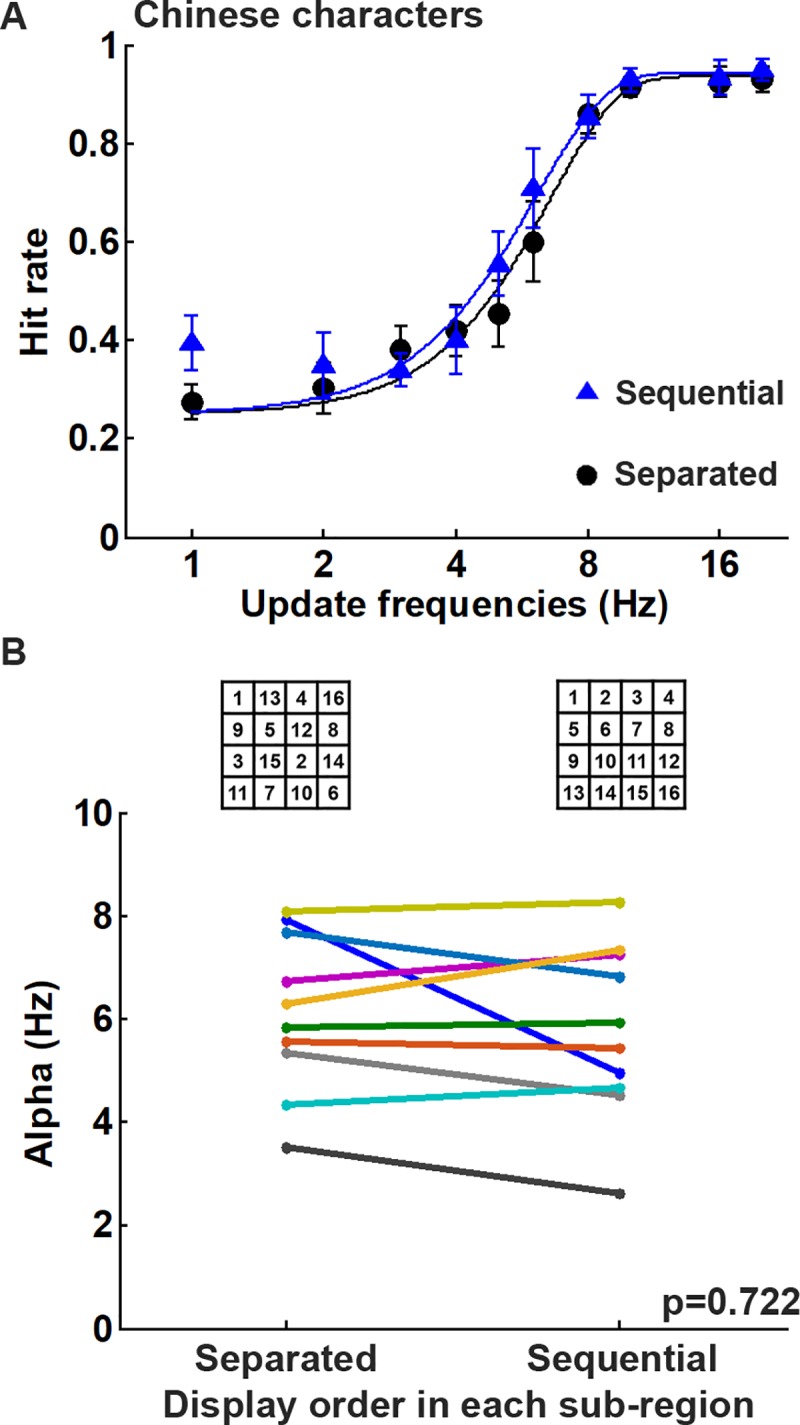
The performance using different display orders in each sub-region using DPSS is not significantly different. (A) Average hitting rates and fitting curves at the different update frequencies under the DPSS conditions using spatially separated and sequential phases. The experiments were performed in a dark room at 8° visual angle, and the DPSS condition involved 16 divisions. (B) Comparisons of the alpha values obtained by fitting to the psychometric function for each subject showed that there was no difference in performance with respect to the display order in each sub-region when carrying out the Chinese character recognition tasks (*p* = 0.722, n = 10).

To examine the effect of flash order in a sub-region of the DPSS display on visual persistence, the Chinese character recognition task was carried out using both spatially separated and spatially sequential flash orders (Fig 3). It was found that the flash order in a sub-region of the DPSS display had little effect on the character recognition tasks (Fig 8). On average, subjects were able to recognize the Chinese characters with 62.5% hit rate at an update frequencies of 6.02 Hz and 5.52 Hz when the task was performed using separated and sequential flash orders, respectively (Fig 8A). When the alpha values were compared between the display patterns at different flash orders in a sub-region of the DPSS display (Fig 8B), there was no significant difference between two flash orders for the Chinese character recognition task (*p* = 0.722; n = 10). This result suggests that visual performance during character recognition is not influenced by the switching order of multiple frames.

### Experiment 6: Effect of DPSS division number

**Fig 9:**
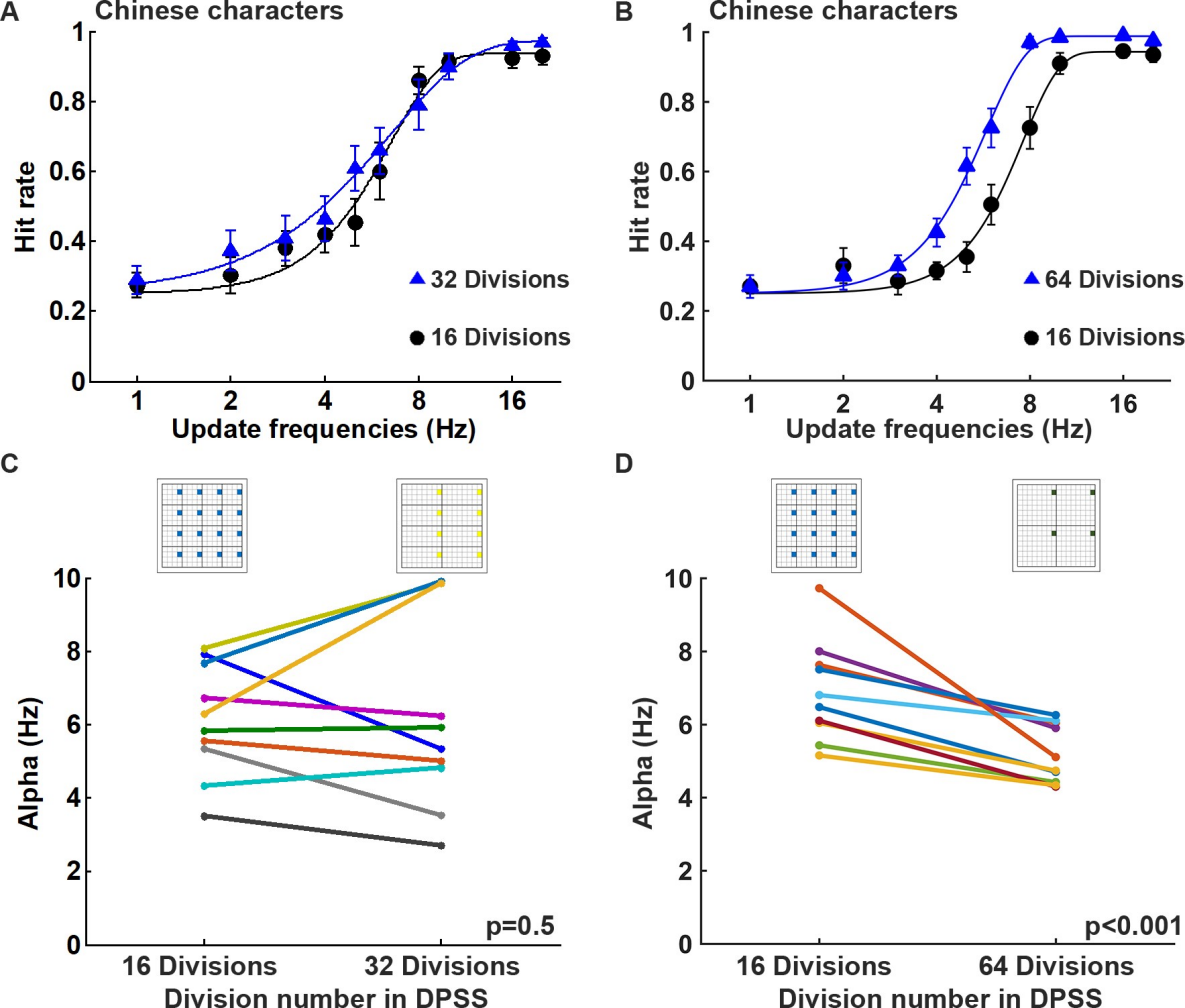
The division number of the DPSS is able to affect performance during Chinese character recognition in negative contrast. (A)&(B) Average hitting rates and fitting curves at different update frequencies using 16 *vs*. 32 divisions and 16 *vs*. 64 divisions, respectively, during negative contrast Chinese character recognition. Both experiments were performed at 8° visual angle and in a dark room, while the DPSS condition was using spatially separated phases. (C)&(D) The comparisons of the alpha values obtained by fitting to the psychometric function for each subject showed that the performance of 16 vs. 32 divisions in the Chinese character recognition task was not significantly different, but the performance of 16 *vs*. 64 divisions in the Chinese character recognition task was significantly different (16 *vs*. 32 divisions, *p* = 0.5, n = 10; 16 *vs*. 64 divisions, *p* < 0.001; n = 10).

To examine the effect of DPSS division number on visual persistence, the Chinese character recognition task was conducted using 16 divisions (16 sub-regions in the 16x16 array), 32 divisions (8 sub-regions in the 16x16 array), and 64 divisions (4 sub-regions in the 16x16 array) of the DPSS display (Fig 3). Two experiments of 16 *vs*. 32 and 16 *vs*. 64 divisions was performed separately. It was found that the DPSS division number had little effect on the character recognition task of 16 *vs*. 32, but the DPSS division number had a significantly different effect on the character recognition task of 16 *vs*. 64 (Fig 9). On average, subjects were able to recognize the Chinese characters with 62.5% hit rate at the update frequencies of 6.02 Hz and 5.46 Hz when the task was performed using 16 and 32 divisions of the DPSS display, respectively (Fig 9A). However, subjects could recognize the Chinese characters with 62.5% hit rate at the update frequencies of 7.11 Hz and 5.21 Hz when the task was performed using 16 and 64 divisions of the DPSS display, respectively (Fig 9B). When the alpha values were compared between the display patterns with different division numbers of the DPSS display, there was no significant difference between 16 and 32 divisions for the Chinese character recognition task (Fig 9C; *p* = 0.500; n = 10). Surprisingly, the alpha values between 16 and 64 divisions for the Chinese character recognition task was significant different (Fig 9D; *p* < 0.001; n = 10). These findings indicate that visual performance during character recognition is affected by the DPSS division number.

## Discussion

### Negative contrast pattern recognition requires faster temporal integration

Patterns with a negative contrast are common in daily life. For example, words in a newspaper and on a computer screen are shown as dark objects against a light background. Thus, when designing a retinal prosthesis, it is important to test visual perception using patterns with both a negative and a positive contrast. Unfortunately, most previous studies targeting prosthetics implanted patients only tested pattern recognition using positive contrast [22]. During the DPSS design approach, it was found that the Chinese and Korean characters with a negative contrast require a higher update frequency to recognize them accurately than those that have a positive contrast (Fig 4). For pattern recognition in DPSS, due to the limits imposed by visual persistence, subjects may not integrate all of the dots in a character when performing the task. Our results are similar to the previous study in which subjects were able to recognize Chinese characters even when only a partial pattern is displayed [23]. Specifically, in the previous study, recognition accuracy fell below 80% when there was a dropout of dots greater than 20% and the accuracy was below 50% when there was a 40% dropout. Since the number of the bright dots in negative contrast is much higher than in positive contrast (190 vs. 68 dots on average; Table 1 and Fig 2), the number of dropout pixels in negative contrast will be significantly higher than in positive contrast at similar update frequencies. This makes character recognition under negative contrast conditions more difficult than under positive contrast conditions. Thus, negative contrast patterns will require a higher update frequency when DPSS is implemented and faster temporal integration during perception in order to obtain the same pattern recognition accuracy as the obtained with a similar positive contrast pattern.

### Free eye movement improves visual persistence in pattern recognition

Although the subjects had to stare at the center of the display board at the beginning of the task, they were not asked to fixate on the pattern throughout the experiment. Thus, there were saccadic eye movements during the display period (1 sec), and subjects could use only central vision to recognize the pattern. As a consequence, the current study cannot delineate any effect of the peripheral vision to the recognition results. Nevertheless, this free eye movement seems to help the subjects to spatially and temporally integrate Chinese and Korean characters accurately, regardless visual angles (8°, 2°, and 1°) of the display (Fig 5). Moreover, previously it has been shown that reading accuracy is not significant affected for the Chinese character sizes at angles of 3°x3°, 5°x5°, and 7°x7° [23]. Taken these findings together, it suggests that a retinal prosthesis with a DPSS design should be able to have the center-to-center distance of the electrodes improved to about 0.0625° (the 16x16 pixels array covers around 1° of the visual angle in the human retina); this could potentially increase the visual acuity provided by the prosthesis via an increase in the electrode density.

### Display brightness rather than ambient light level affects visual persistence in pattern recognition

Previous studies have reported that ambient light intensity contributes to the performance when visual persistence is in play [1, 5, 24]. Different from this general principle, the current findings show that pattern recognition when a DPSS approach is used is not affected by the background light levels (Fig 6). One possible reason for this is that the light level in the test room (85 lux) was too low to affect visual persistence [24], while at the same time the intensity of the LED display board (20 lux) was relatively high. These conditions seem to allow the display to dominate during the visual recognition tasks in both the dark and the light environments. However, the pattern contrast inevitably decreases as the background illumination increases (the pedestal effect), thus the contrast sensitivity must be affected at different ambient light levels [25, 26]. Nevertheless, the high intensity of the display board would seem to have overcome sensitivity to the pedestal effect and allowed visual persistence to function independently of ambient light intensity. Consistent with this explanation, our result also showed that the performance of Chinese character recognition was improved when the display brightness was reduced to 0.25% of the original intensity in the dark room (Fig 7). It has been suggested that the critical fusion frequency depends on the luminance of the stimulus [27]. When the display brightness level was significantly low, switching of light among different pixels in DPSS mode became less detectable and the image appeared more stable at relatively lower update frequency. These results suggest that display brightness rather than ambient light level affects visual persistence in pattern recognition. It also implies that balance between the stimulation strength and ambient light level is an important factor for optimal DPSS retinal prosthesis design.

### The effect of the flash order of the 4x4 sub-region on visual persistence during pattern recognition

Although the current findings indicate that the flash order of the 4x4 sub-regions do not affect performance during Chinese character recognition (Fig 8), a pilot study using a sequential flash order involving 1x16 sub-regions (the pattern was displayed row-by-row, similar to the scanning CRT monitor in a low frequency) showed that this task was much easier than a sequential flash order in the 4x4 sub-region. This difference suggests that continuously scanning the character horizontally or vertically is able to facilitate pattern recognition by using short-term memory [5]. Even though the sequential order in the 4x4 sub-regions also require subjects to scan the character continuously, the sequence is interrupted every four phases because of the row switch (Fig 3). This feature may explain our result wherein the sequential order was not better than the separated order using 4x4 sub-region DPSS (Fig 8). Moreover, the sequential order display may cause cross-talk between adjacent pixels as two neighbor electrodes might interact with the same group of neurons if stimulated sequentially [28]. Previous studies have also reported that the responses of retinal ganglion cells (RGCs) are desensitized after the first pulse, and this reduction in response increases more rapidly at higher stimulation frequencies [29–31]. As a result, the separated order was designed in the present study to reduce the effect of cross-talk by the use of spatial separation (increasing the distance between the electrodes of two consecutive phases), and this should reduce the effect of the RGC desensitization by providing temporal separation (increasing the time interval between two neighbor electrode stimulations).

### Visual persistence depends on the time required for temporal integration and DPSS divisions

When the 16, 32, and 64 division numbers of DPSS were running at the same update frequency, the phase number of each cycle and the active pixels of each phase were different. If the visual system integrates the information by combining all pixels from all phases, the 32 and 64 divisions should provide twice and four-times as much information as the 16 divisions at any moment in time, respectively. However, the current finding showed that there was no significant difference between the performances using 16 and 32 divisions (Fig 9A & 9C). Thus, this result may suggest that visual integration is determined by the time length of visual persistence rather than the amount of visual information. Interestingly, our result showed that 64 divisions of DPSS improved the performance of Chinese character recognition when compared with 16 divisions (Fig 9B & 9D). It should be noted that both 32 and 64 divisions provide less perceived pixels than 16 divisions at any moment in time, thus less display brightness. Based on the result of the present study (Fig 7) that display brightness affects the pattern recognition task, it is conceivable that subjects could recognize the pattern shown on 64 divisions at lower update frequency, as a result of decreasing the critical flicker frequency at lower image brightness. Thus, this finding suggests that implementing a DPSS using higher divisions has great potential and should further increase the power efficiency of a retinal prosthesis design. Despite this seemingly advantage of higher DPSS divisions in power efficiency improvement, there is an upper limit of division numbers in the retinal prosthesis design. Since the maximal output voltage of the retinal chip is fixed by design [14], increasing DPSS divisions inevitably will exceed the limit of the output voltage, thus offset the intended power increase.

### DPSS has the additional benefit of reducing the pulse width in retinal prosthesis

By concentrating the power from all 16 photovoltaic units at any moment in time, the DPSS approach will significantly increase the power efficiency by sixteen times (Fig 1). The pulse width of each phase of electrical stimulation is fixed by the DPSS design. Ideally, if one increases the pulse width 16 times at each electrode without implementing the DPSS design, there may also be a gain via an increase in power efficiency of sixteen times. However, it is known that the threshold of RGC responses upon electrical stimulation are relatively constant only when there is short electrical pulse stimulation [32, 33]. A previous study has also reported that the threshold increased when the pulse width was beyond 1 ms [15]. Therefore, the DPSS design has the additional advantage of keeping the pulse short and lowering the threshold of the RGCs during electrical stimulation.

### Visual persistence in patients with retinal prosthesis

A prolonged visual representation begins with activation of photoreceptors in the retina [34]. However, patients with RP and AMD lose their vision due to photoreceptor degeneration [35, 36], and thus it could be argued that implementing DPSS for a retinal prosthesis may not work for RP and AMP patients. Nevertheless, it has been known that visual persistence exists at other levels of the visual pathway [34, 37]. For example, it has been reported that patients implanted with a subretinal prosthesis could perceive letters when a sequential activation of 4x4 electrodes is implemented [38]. This indicates that persistence of information may also be mediated in visual cortex and/or other higher-level brain areas. Thus, it is likely that the RP and AMD patients will benefit from the DPSS design proposed in the present study based on visual persistence beyond the photoreceptor level. A different study reported that patients implanted with epiretinal prosthetics could easily discriminate spatially identical patterns generated by synchronous or asynchronous pulse train stimuli across groups of four electrodes [39], the reason might be that the interaction between electrodes could influence subjective brightness perception and that the interaction depended on the electrode pair distance and time shift [40]. In the present study, all of our subjects could perceive light spots clearly, and we did not observe any interaction when the temporal frequency and electrode pair distance varied. However, it is known that the fading effect could reduce the perceived brightness and result in a poorly localized percept after the stimulus offset [41]. Thus, it may have a negative impact on visual pattern reconstruction if the perception is different from phase to phase. Nevertheless, it suggests that a better understanding of spatiotemporal effect of visual persistence from clinical studies in developing retinal prosthesis with DPSS technology is much needed in the future.

## Supporting information

S1 Fig The visual stimuli used during the non-repeated pattern recognition task. All 210 pixelized images of the Chinese characters in negative contrast.
(TIF)

S2 Fig The performance of Chinese character recognition task with non-repeated and repeated characters using DPSS is not significantly different. (A) Average hitting rates and fitting curves at the different update frequencies in both non-repeated and repeated Chinese character recognition task. The experiments were performed in a dark room at 8° visual angle, and the DPSS condition was 16 divisions with spatially separated phases. (B) The comparisons of the alpha values obtained by fitting to the psychometric function for each subject showed that the performance of non-repeated and repeated Chinese character recognition task was not significantly different (*p* = 0.461, n = 10).
(TIF)

S3 Fig The response time of performing Chinese character recognition task at various update frequencies. The median of response time for making a choice during the Chinese character recognition task at various DPSS update frequencies. Average hitting rates and a fitting curve at the different update frequencies during the Chinese character recognition task were also shown. The experiment was performed in a dark room at 8° visual angle, and the DPSS condition was 16 divisions with spatially separated phases.
(TIF)

S1 Data OriginalData. (XLSX)
