# Supplementary figures and images for: Spatiotemporal integration of visual stimuli and its relevance to the use of a divisional power supply scheme for retinal prosthesis
Source: PLoS One. 2020 Feb 21;15(2):e0228861. doi: 10.1371/journal.pone.0228861 (PMC7034871; doi:10.1371/journal.pone.0228861)

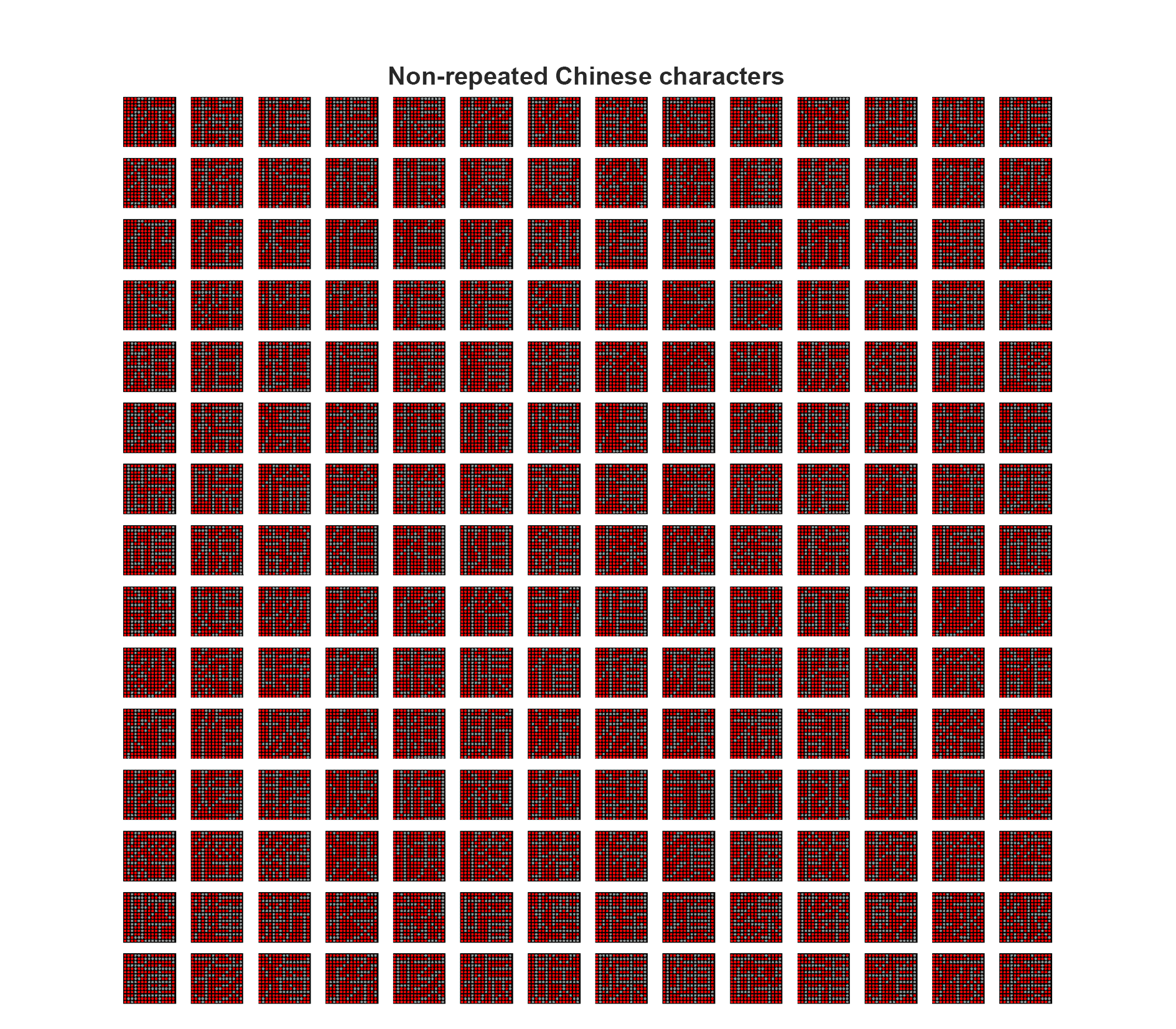

Supplement: S1 Fig — All 210 pixelized images of the Chinese characters in negative contrast. (TIF) [file pone.0228861.s001.tif]

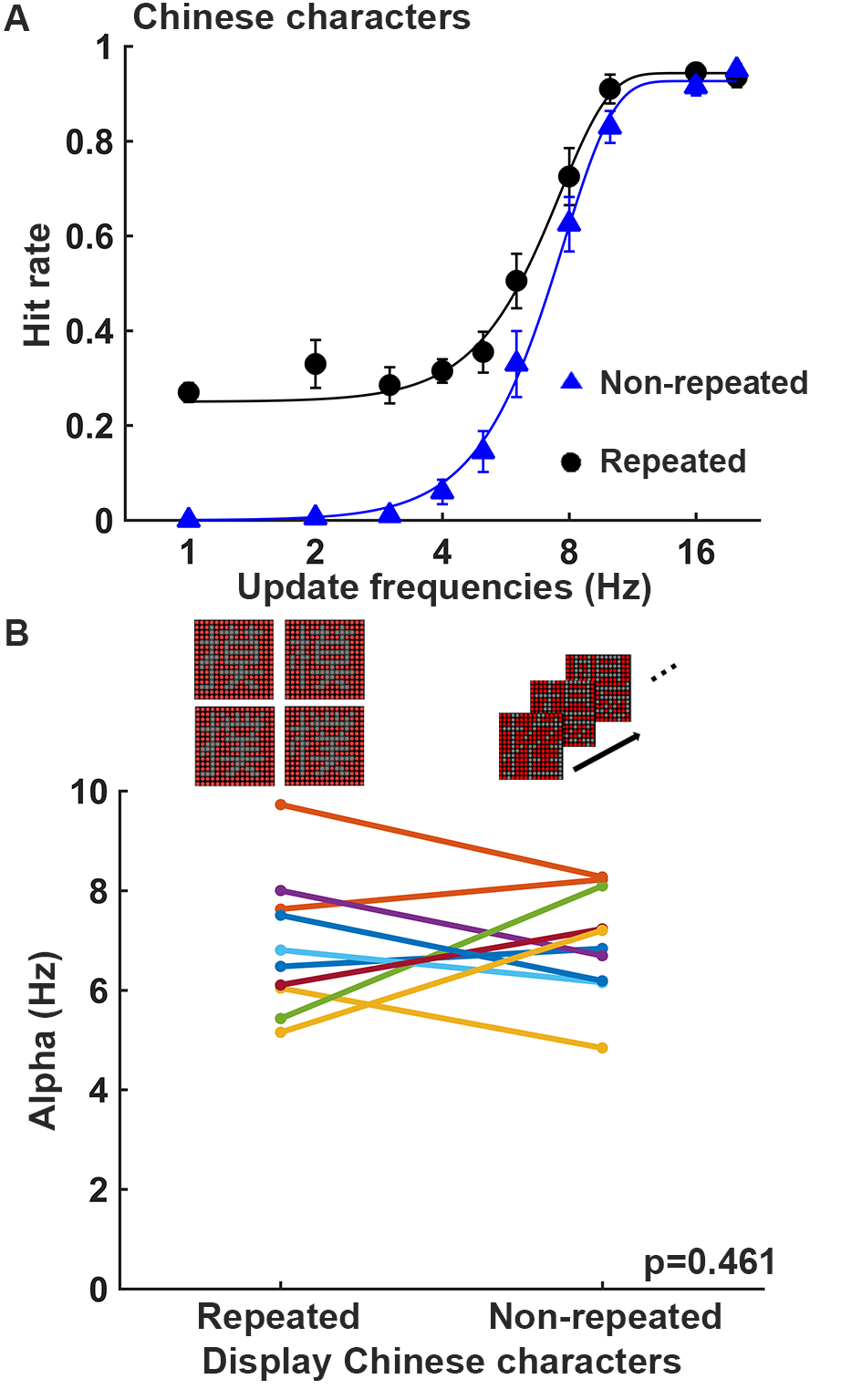

Supplement: S2 Fig — (A) Average hitting rates and fitting curves at the different update frequencies in both non-repeated and repeated Chinese character recognition task. The experiments were performed in a dark room at 8° visual angle, and the DPSS condition was 16 divisions with spatially separated phases. (B) The comparisons of the alpha values obtained by fitting to the psychometric function for each subject showed that the performance of non-repeated and repeated Chinese character recognition task was not significantly different (p = 0.461, n = 10). (TIF) [file pone.0228861.s002.tif]

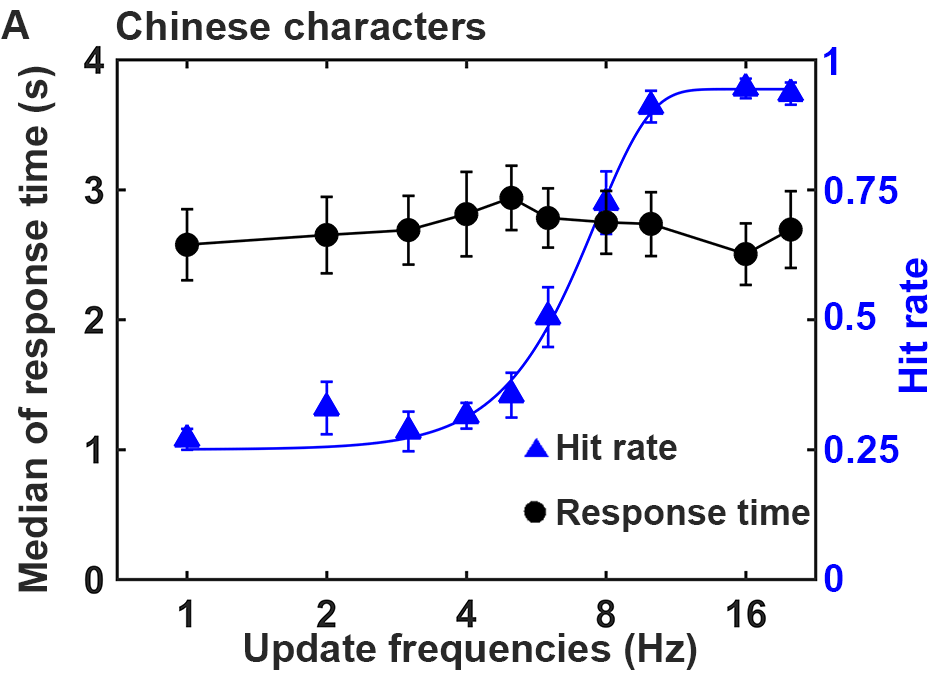

Supplement: S3 Fig — The median of response time for making a choice during the Chinese character recognition task at various DPSS update frequencies. Average hitting rates and a fitting curve at the different update frequencies during the Chinese character recognition task were also shown. The experiment was performed in a dark room at 8° visual angle, and the DPSS condition was 16 divisions with spatially separated phases. (TIF) [file pone.0228861.s003.tif]
